# Feasibility of a Cinematic–Virtual Reality Program Educating Health Professional Students About the Complexity of Geriatric Care: Pilot Pre-Post Study

**DOI:** 10.2196/64633

**Published:** 2025-02-12

**Authors:** Elizabeth A Beverly, Samuel Miller, Matthew Love, Carrie Love

**Affiliations:** 1 Department of Primary Care, Ohio University Heritage College of Osteopathic Medicine, 1 Ohio University, Athens, OH, 45701, United States, 1 7405934616, 1 740-593-2205; 2 The Diabetes Institute, Ohio University, Athens, OH, United States; 3 Department of Medicine, Ohio University Heritage College of Osteopathic Medicine, Athens, OH, United States

**Keywords:** virtual reality, VR, aging, geriatric syndromes, diabetes, elder abuse and neglect, gerontology, geriatrics, older, elderly, education, student, cinematic, video, head mounted, feasibility, experience, attitude, opinion, perception, elder abuse, chronic conditions, older adult care, health intervention, randomized controlled trial

## Abstract

**Background:**

The US population is aging. With this demographic shift, more older adults will be living with chronic conditions and geriatric syndromes. To prepare the next generation of health care professionals for this aging population, we need to provide training that captures the complexity of geriatric care.

**Objective:**

This pilot study aimed to assess the feasibility of the cinematic–virtual reality (cine-VR) training in the complexity of geriatric care. We measured changes in attitudes to disability, self-efficacy to identify and manage elder abuse and neglect, and empathy before and after participating in the training program.

**Methods:**

We conducted a single-arm, pretest-posttest pilot study to assess the feasibility of a cine-VR training and measure changes in attitudes to disability, self-efficacy to identify and manage elder abuse and neglect, and empathy. Health professional students from a large university in the Midwest were invited to participate in 1 of 4 cine-VR trainings. Participants completed 3 surveys before and after the cine-VR training. We performed paired *t* tests to examine changes in these constructs before and after the training.

**Results:**

A total of 65 health professional students participated in and completed the full cine-VR training for 100% retention. Participants did not report any technological difficulties or adverse effects from wearing the head-mounted displays or viewing the 360-degree video. Out of the 65 participants, 48 completed the pre- and postassessments. We observed an increase in awareness of discrimination towards people with disability (*t*_47_=−3.97; *P*<.001). In addition, we observed significant improvements in self-efficacy to identify and manage elder abuse and neglect (*t*_47_=−3.36; *P*=.002). Finally, we observed an increase in participants’ empathy (*t*_47_=−2.33; *P*=.02).

**Conclusions:**

We demonstrated that our cine-VR training program was feasible and acceptable to health professional students at our Midwestern university. Findings suggest that the cine-VR training increased awareness of discrimination towards people with disabilities, improved self-efficacy to identify and manage elder abuse and neglect, and increased empathy. Future research using a randomized controlled trial design with a larger, more diverse sample and a proper control condition is needed to confirm the effectiveness of our cine-VR training.

## Introduction

The US population is aging. Over the next 3 decades, the number of Americans aged 65 and older is projected to increase from 58 million in 2022 to 82 million in 2050, representing a 47% increase [1]. With this demographic shift, more older adults will be living with chronic conditions. According to the National Council on Aging, 94.9% of adults aged 60 years and older have at least one chronic condition and 78.7% have two or more [2]. Furthermore, 42% of adults aged 60 years and older have obesity, which increases the risk for cardiovascular disease, type 2 diabetes, and certain cancers [3]. This translates to older Americans requiring more health care and access to health care professionals. Thus, training the next generation of health care professionals in the complexity of care for older adults is an urgent need.

Despite this need, the education system lacks adequate training in geriatric care [4]. Health professional training should address age-related physiological, physical, cognitive, and psychosocial changes that may negatively impact quality of life, disability, morbidity, and mortality [5,6]. Furthermore, this training should emphasize the early detection of and treatment for geriatric syndromes (eg, polypharmacy, falls, frailty, and incontinence) given their high prevalence and frequent under-recognition in older adults [7]. Finally, health professional training should draw attention to implicit and explicit biases in aging as well as ethical issues related to elder abuse and neglect and end-of-life care [8].

Clinical simulation may be an innovative approach to teaching health professional students about the complexity of care in older adults. Previous research demonstrates that clinical simulation is an effective methodology for health professional students [9]. The value of clinical simulation is that it replaces or enhances real experiences with guided experiences that replicate the real world in a fully interactive manner [10]. Furthermore, clinical simulation is repeatable, which allows for the repetition of acquired skills with appropriate feedback [11]. Clinical simulation can be delivered in different modalities, including role play, the use of standardized patients, computer screen-based simulation, and the use of electronic patient records [11]. Electronic patients are most commonly mannequin-based or replicas of clinical sites; however, new advancements in technology have led to more sophisticated and realistic virtual reality (VR) based simulations [12].

Cinematic-VR (cine-VR) is one type of VR-based simulation. Specifically, cine-VR leverages 360-degree video and spatialized audio through film. It differs from traditional VR, which uses computer-generated characters and worlds. In cine-VR, filmmakers apply the techniques of cinema (ie, narrative storytelling, script writing, actors, lighting, and postproduction) to create an immersive, visually compelling environment [13,14]. We created a cine-VR training program to depict the complexity of geriatric care. Our clinical simulation depicted an older adult with multimorbidity, disability, and several geriatric syndromes. In addition, we wove in themes of intersectionality with regards to ageism, ableism, and racism. We conducted a pilot study to assess the feasibility and acceptability of the cine-VR training program with health professional students. We also measured changes in health professional students’ attitudes toward disability, self-efficacy to identify and manage elder abuse and neglect, and empathy.

## Methods

### Research Design

We conducted a single-arm, pretest-posttest study to assess changes in attitudes to disability, self-efficacy to identify and manage elder abuse and neglect, and empathy. The pilot study was designed to assess the feasibility of our data collection methods, willingness of participants to complete the cine-VR training program, sample size estimation, and refinement of measurements. We will use the findings to inform the development of a future randomized controlled trial to compare the cine-VR training program to traditional instruction.

### Ethical Considerations

We obtained ethics approval from the Ohio University Office of Research Compliance Institutional Review Board (approval number: #22-X-153). We ensured our pilot study met the requirements set forth in the regulations on public welfare in Part 46 of Title 45 of the Code of Federal Regulations (45 CFR 46) by complying with federal, state, and local laws and regulations for human subjects; the principles set forth in “The Belmont Report,” and the Helsinki Declaration of 1975. All participants provided electronic informed consent. Informed consent was completed before participation in the study.

### Cine-VR Episodes

The cine-VR episodes were designed to educate health professional students about complex health conditions, social drivers of health, and implicit bias. We conveyed these objectives through our patient, John Chen, an 80-year-old Chinese American man with a 14-year history of type 2 diabetes and comorbid hypertension. Mr. Chen is a person who is hard of hearing with a mobility disability. His most recent HBA_1c_ level was 9.7%. His medications include metformin and glyburide for his diabetes and lisinopril for his blood pressure. He also has osteoarthritis, decreased renal function (eGFR=58 mL/min/1.73 m^2^), elevated triglycerides due to his glucose levels (299 mg/dL), and a body mass index of 23.1 kg/m^2^. He has not received diabetes self-management education and support, despite being enrolled in both Medicare and Medicaid. Recently, Mr Chen moved in with his son’s family, but he is having a difficult time adjusting to his new home. Although he is able to move around on his own, his ambulation is unsteady, and he falls often. He also experiences frequent urinary incontinence, which frustrates his son and daughter-in-law because they have to clean him. Furthermore, Mr Chen does not drive, shop, cook, or bathe on his own. Therefore, he is reliant on his family to meet most of his daily needs. This leaves Mr Chen feeling isolated, dependent, and ashamed of his physical limitations.

In the cine-VR training program, participants watched 6 episodes, ranging in length from 2 minutes to 5 minutes. The episodes captured 3 separate patient-provider interactions, including visits with a primary care physician, an urgent care physician, and an emergency department physician. Participants were encouraged to identify issues with his medical care as well as concerns about the caregiving he received at home. Specifically, participants were challenged to figure out why Mr Chen was experiencing so many falls, injuries, and health emergencies.

The fifth and sixth episodes of the cine-VR training program were “guided simulations,” or prerecorded cine-VR face-to-face conversations with the emergency department physician and Mr Chen. The guided simulations were designed to simulate a high-stakes conversation, to give participants an opportunity to practice difficult conversations without the pressure of causing harm. Participants were instructed to speak predetermined dialogue that appeared at the bottom of the 360-degree video visible within the headset. The first “guided simulation” was a conversation between the participant and the emergency department physician. The participant assumed the role of a clinical colleague and encouraged the physician to inquire further about the bruises observed on John’s body and to call social work for suspicion of elder abuse and neglect. The second “guided simulation” was a conversation between the emergency department physician, Mr Chen, and a social worker. In this “guided simulation,” the participant took on the role of the emergency department physician and asked Mr Chen difficult questions about his injuries, his family, and life at home.

### Cine-VR Training Curricular Content

We developed curricular content to be taught in tandem with the cine-VR episodes. The curriculum included 6 debriefs or reflections that reinforced key takeaways from each cine-VR episode. The key takeaways from each episode focused on the following content: (1) Diabetes, disability, and the aging population, (2) bias toward disability, (3) association between disability and elder abuse and neglect, (4) recognizing elder abuse and neglect, (5) identifying risk factors for elder abuse and neglect, and (6) reporting elder abuse and neglect. An experienced behavioral diabetes researcher (EAB) delivered both trainings. The integrity of the curricular content was ensured by written materials, a peer-review process of all materials, delivery by one trained behavioral diabetes expert, and team member observation of cine-VR training.

### Cine-VR Technology

Participants viewed the cine-VR episodes with Pico G2 4K head-mounted displays. These head-mounted displays allowed participants to move their head and body in any direction to choose what aspects they paid attention to during each cine-VR episode. We also synchronized all cine-VR episodes from a central computer using VR Sync software so all participants viewed the same content in the head-mounted display at the same time.

### Power Analysis

This was a pilot study so we did not conduct an a priori power analysis. Using the guidance of Lancaster et al [15], we recruited a minimum sample size of 30 participants.

### Recruitment

Health professional students were recruited from a large university in the Midwest. Eligibility criteria for participating in the pilot study included English-speaking and reading adults aged 18 years and older who were enrolled during the 2022‐2023 academic year and majoring in one of the following programs: premedicine program, speech-language pathology, audiology, nutrition and dietetics, physical therapy, nursing (any level), social work (any level), athletic training, exercise physiology, pre-physician assistant program, psychology, public health administration, or public health. There were no other exclusion criteria. Specifically, we hosted 4 in-person cine-VR training sessions on campus in classrooms and invited students from those health professional programs to participate. Sessions were hosted on October 17, 2022, October 24, 2022, April 5, 2023, and June 6, 2023.

### Measures

To assess the feasibility of the cine-VR training program, we measured recruitment, retention, length of time required to recruit, rate of completion of the cine-VR training, and feasibility of the data collection measures. In addition, participants provided demographic factors (age, gender, race, ethnicity, year in program, and program) and completed the following measures:

#### Attitudes to Disability Scale

The Attitudes to Disability Scale [16] is a 16-item validated scale that assesses attitudes toward disability and attitudes toward people with a disability. The 16 items were answered on a 5-point Likert scale ranging from 1 (strongly disagree) to 5 (strongly agree). The scale included 4 domains or subscales with good internal consistency: Inclusion—focuses on attitudes toward the inclusion and exclusion for people with disabilities as well as the burden on families and society (Cronbach *α*=0.76), Discrimination—focuses on awareness of unfair and prejudicial treatment to people with disabilities (Cronbach *α*=0.74), Gains—focuses on positives of having a disability (Cronbach *α*=0.75), and Prospects—focuses on future hopes and whether disability impacts those hopes (Cronbach *α*=0.72) [16].

#### Responding to Elder Abuse in GERiAtric Care—Provider Questionnaire

The Responding to Elder Abuse in GERiAtric Care—Provider Questionnaire [17] is a validated 25-item scale that assesses health care provider preparedness to identify and manage elder abuse and neglect. For the purposes of this study, we used 8 of the 25 items. The items we used addressed self-efficacy to identify and manage elder abuse and neglect on a 0 to 10 scale. The 17 excluded items were written for practicing health care professionals. In our sample, the 8 self-efficacy questions demonstrated excellent internal consistency for identifying and managing elder abuse and neglect (Cronbach *α*=0.95) [17].

#### Jefferson Scale of Empathy Health Professionals Students Version

The Jefferson Scale of Empathy Health Professionals Students Version [18] is a 20-item validated scale that assesses empathy among health professional students. The 20 items were answered on a 7-point Likert scale, ranging from 1 (strongly disagree) to 7 (strongly agree). Scores are summed for a composite score, ranging from 20 to 140. The scale demonstrated good internal consistency (Cronbach *α*=0.85) [18].

### Data Collection

Participants completed the assessments through Qualtrics, an electronic questionnaire service. To access the preassessment, participants scanned a QR code from a Microsoft PowerPoint slide that directed them to the Qualtrics website. The Qualtrics website included a brief description of the study, the informed consent form, demographic questions, and the 3 measures. All participants provided informed consent without a signature in Qualtrics before completing the measures. After the cine-VR training, participants scanned a different QR code that directed them to the postassessment measures.

We collected data anonymously. To link participants’ pre- and postassessment responses, we included 3 questions at the beginning of the pre- and postassessments, which served as a unique identifier (ie, model of first car, high school mascot, and the day of the month on which they were born [eg, 31]). Total time to complete the pre- and postmeasures took approximately 15‐20 minutes. Participants received a US $25 gift card for human subject compensation. To maintain anonymity, participants clicked on a new Qualtrics link that was not connected to their pre- or postmeasures to receive a gift card; it was possible that participants received compensation without completing all pre- and postmeasures.

### Statistical Analysis

First, we assessed participants’ demographic factors using descriptive statistics and presented them as means and SDs or sample size and percentages. Next, we examined the distribution of the data to ensure they met statistical test assumptions. To assess changes pre- and post–cine-VR training, we conducted paired *t* tests; all test statistics are presented with α values and degrees of freedom. We also calculated effect sizes using Cohen *d*, with a small effect=0.2, medium effect=0.5, and a large effect=0.8. We defined statistical significance as a *P* value less than .05 and conducted analyses in IBM SPSS statistical software (version 29.0).

## Results

### Feasibility

A total of 65 health professional students participated in and completed the full cine-VR training for 100% retention. Participants did not report any technological difficulties or adverse effects from wearing the head-mounted displays or viewing the 360-degree video. We scheduled 4 cine-VR trainings in a 6-month period to recruit the 65 participants, and all 4 trainings lasted approximately 60 minutes. For data collection, 65 participants completed the preassessment measures; however, only 48 completed the postassessment measures, resulting in a 74% completion rate. The 74% rate of completion suggests that we may want to consider adaptations or refinements to our selected measures. Overall, the ease of recruitment, length of time required to recruit, retention in the cine-VR trainings, and feasibility of data collection methods suggest that the cine-VR training was feasible.

### Demographics

A total of 48 health professional students consented to participate in the pilot study and completed all pre- and postassessments. The mean age of participants was 22.5 (SD 3) (see Table 1). A total of 41 participants (85.4%) self-identified as women, 6 (12.5%) self-identified as men, and 1 (2.1%) self-identified as nonbinary. The participants self-identified their race as follows: 6.3% (3/48) Asian or Pacific Islander, 18.8% (9/48) Black or African American, 4.2% (2/48) Mixed Race, and 70.8% (34/48) White. For ethnicity, 2 participants (4.2%) self-identified as Hispanic, Latino, or Spanish origin and 2 (4.2%) self-identified as Mexican, Mexican American, or Chicano. The majority of participants were in the final year of their program (37.5%, 18/48), with premedicine as the most reported major (42.6%, 20/48).

**Table 1.** Participant demographic characteristics in the cinematic-virtual reality (cine-VR) study (N=48).

Variable	Results
Age (years), mean (SD)	22.5 (3)
**Gender, n (%)**	
Woman	41 (85.4)
Man	6 (12.5)
Nonbinary	1 (2.1)
Transgender	0 (0)
Genderqueer	0 (0)
An identity not listed	0 (0)
**Ethnicity, n (%)**	
Hispanic, Latino, or Spanish origin	2 (4.2)
Mexican, Mexican American, or Chicano	2 (4.2)
Non-Hispanic, Non-Latino, or Non-Spanish	44 (91.6)
**Race, n (%)**	
American Indian or Pacific Islander	0 (0)
Asian or Pacific Islander	3 (6.3)
Black or African American	9 (18.8)
Middle Eastern	0 (0)
Mixed Race	2 (4.2)
White	34 (70.8)
**Program** ^**a**^ **, n (%)**	
Child Life Specialist	18 (38.3)
Exercise Physiology	1 (2.1)
Nutrition	4 (8.5)
Pre-Medicine	20 (42.6)
Pre-Physician Assistant	2 (4.3)
Psychology	2 (4.3)
**Year in Program** ^**b**^ **, n (%)**	
Year 1	9 (18.8)
Year 2	10 (20.8)
Year 3	9 (18.8)
Year 4	18 (37.5)

a Missing values “Program” (n=1).

b "Year in Program” (n=2).

### Attitudes Toward Disability Findings

Pre- and postdomain scores for the Attitudes to Disability Scale can be found in Table 2. Post–cine-VR training, we observed significant changes in 1 of the 4 Attitudes to Disability Subscales: Discrimination Domain (*t*_47_=−3.97, *P*<.001). This change had a Cohen *d*=0.55, indicating a medium effect. This finding suggests that the cine-VR training may have increased participants’ awareness of discrimination towards people with a disability. In the Discrimination Domain, 3 of the 4 items showed a significant change with more participants “agreeing” and “strongly agreeing” with the following statements posttraining: “People often make fun of disabilities” (*t*_47_=−3.92; *P*<.001); “People tend to become impatient with those with a disability” (*t*_47_=−3.19; *P*=.003); and “People tend to treat those with a disability as if they have no feelings” (*t*_47_=−2.69; *P*=.01). Post-cine-VR training, we did not observe significant changes in the Inclusion Domain (*t*_47_=−.16; *P*=.88), Gains Domain (*t*_47_=−1.76; *P*=.08), or Prospects Domain (*t*_47_=−.30; *P*=.77).

**Table 2.** Participants’ attitudes to disability scale mean scores before and after the cinematic-virtual reality (cine-VR) training program (N=48).

Questions	Pre-VR^a^, mean (SD)	Post-VR, mean (SD)	*t* test (*df*)	*P* value
**Inclusion domain**				
People with a disability find it harder than others to make new friends.	3.9 (0.6)	3.8 (0.8)	.948 (47)	.35
People with a disability have problems getting involved in society.	3.5 (1.2)	3.8 (0.8)	−2.047 (47)	.046
People with a disability are a burden on society.	1.2 (0.4)	1.3 (0.6)	−1.000 (47)	.32
People with a disability are a burden on their family.	1.9 (0.9)	1.7 (1.0)	1.944 (47)	.06
Total	2.6 (0.6)	2.6 (0.5)	−.156 (47)	.88
**Discrimination domain**				
People often make fun of disabilities.	3.9 (0.6)	4.2 (0.6)	−3.923 (47)	<.001
People tend to become impatient with those with a disability.	3.9 (0.8)	4.3 (0.7)	−3.186 (47)	.003
People tend to treat those with a disability as if they have no feelings.	3.5 (1.1)	3.9 (0.8)	−2.687 (47)	.01
People with a disability are easier to take advantage of (exploit or treat badly) compared with other people.	3.7 (0.9)	3.9 (1.0)	−1.095 (47)	.28
Total	3.8 (0.6)	4.1 (0.6)	−3.967 (47)	<.001
**Gains domain**				
Having a disability can make someone a stronger person.	3.8 (0.7)	3.9 (0.8)	−1.219 (47)	.23
Having a disability can make someone a wiser person.	3.7 (0.8)	3.7 (0.8)	.227 (47)	.82
Some people achieve more because of their disability (eg, they are more successful).	3.0 (0.8)	3.2 (0.8)	−1.741 (47)	.09
People with a disability are more determined than others to reach their goals.	3.0 (0.7)	3.1 (0.6)	−1.401 (47)	.17
Total	3.3 (0.5)	3.5 (0.6)	−1.764 (47)	.08
**Prospects domain**				
Sex should not be discussed with people with disabilities.	1.6 (0.9)	1.4 (0.7)	1.752 (47)	.09
People should not expect too much from those with a disability.	1.5 (0.5)	1.6 (0.8)	−1.182 (47)	.24
People with a disability should not be optimistic (hopeful) about their future	1.3 (0.7)	1.5 (0.9)	−1.543 (47)	.13
People with a disability have less to look forward to than others.	1.6 (0.7)	1.6 (0.7)	.423 (47)	.67
Total	1.5 (0.5)	1.5 (0.6)	−.300 (47)	.77

a VR: virtual reality.

### Self-Efficacy to Identify and Manage Elder Abuse and Neglect Findings

Post—cine-VR training, we observed significant changes in participants’ self-efficacy to identify and manage elder abuse and neglect (*t*_47_=−3.36, *P*=.002, Table 3). This change had a Cohen *d*=0.49, approaching a medium effect.

**Table 3.** Participants’ perceived self-efficacy for identifying and managing elder abuse and neglect before and after the cinematic-virtual reality (cine-VR) training program (N=48).

Questions	Pre-VR,^a^ mean (SD)	Post-VR, mean (SD)	*t* test (*df*)	*P* value	Cohen *d*
Asking questions about abuse to an older patient who has clear indications of now being, or having previously been, subjected to abuse.	6.2 (2.4)	7.2 (2.4)	−3.354 (47)	.002	.48
Asking questions about abuse to an older patient who has no clear indications of now being or having previously been, subjected to abuse.	5.7 (2.6)	5.9 (2.4)	−.585 (47)	.56	.08
Ensuring you are able to ask questions about abuse in private to an older patient who has a relative who insists on being present during all contact.	6.1 (2.7)	7.0 (2.4)	−2.911 (47)	.005	.42
In conversation, providing support to an older patient who tells about abuse.	7.4 (2.6)	8.0 (2.1)	−2.438 (47)	.02	.35
Helping an older patient subjected to abuse on to the right body in health care, or to the right support function in society.	6.6 (2.9)	7.7 (2.1)	−3.174 (47)	.003	.46
Helping an older patient subjected to abuse to make a report to the police or social services.	6.9 (2.8)	7.6 (2.2)	−1.984 (47)	.05	.29
Helping and supporting an older patient subjected to abuse, who does not currently want to change his or her situation.	5.5 (2.9)	6.3 (2.6)	−2.433 (47)	.02	.35
Handling the meeting with an older patient who says no to questions about abuse, but where you still have strong suspicions that the patient is subjected to abuse.	5.7 (2.8)	6.6 (2.2)	−2.601 (47)	.01	.38
Total	6.3 (2.4)	7.0 (1.8)	−3.364 (47)	.002	.49

a VR: virtual reality.

In total, 6 of the 8 items demonstrated significant improvements in perceived self-efficacy. Specifically, participants reported increases in their self-efficacy to ask questions about abuse to an older individual who has clear indications of being abused (*t*_47_=−3.354; *P*=.002; Cohen *d*=0.48) and to ensure that they ask questions about abuse in private (*t*_47_=−2.911; *P*=.005; Cohen *d*=0.42). We also observed increases in self-efficacy for providing support to an older individual who discloses abuse (*t*_47_=−2.438; *P*=.02; Cohen *d*=0.35) and directing an older adult experiencing abuse to the right support person or service (*t*_47_=−3.174; *P*=.003; Cohen *d*=0.46). Finally, participants showed improvements in their self-efficacy for helping older adults experiencing abuse who do not want to change their situation (*t*_47_=−2.433; *P*=.02; Cohen *d*=0.35) and handling an older person who says no to questions about abuse despite strong suspicions of abuse (*t*_47_=−2.601; *P*=.01; Cohen *d*=0.38).

One item showed a trend in improving self-efficacy to help an older adult make a report to the police or social services (*t*_47_=−1.984; *P*=.05; Cohen *d*=0.29). Participants did not show an improvement in their self-efficacy to ask questions about abuse to an older adult who has no clear indications of presently or previously experiencing abuse (*t*_47_=−.585; *P*=.56; Cohen *d*=0.08). This finding highlights an area to be addressed in future research.

### Empathy Findings

Pre–cine-VR training empathy scores ranged from 47 to 128, with a mean of 105.7 (SD 16.6). Post–cine-VR training empathy scores ranged from 80 to 131, with a mean of 110.4 (SD 12.1). We observed a significant increase in participants’ empathy scores post–cine-VR training (mean change=4.8; *t*_47_=−2.329; *P*=.02), with a Cohen *d*=0.34 indicating a small effect. This finding indicates a noticeable difference in empathy before and after participating in the cine-VR training. While the effect size is small, an increase in empathy may have practical significance in training health professional students about the complexity of diabetes management. More research is needed to confirm this finding.

## Discussion

### Principal Findings

In this pilot study, we assessed the feasibility of implementing a cine-VR training program with health professional students. Our findings showed that we were able to recruit, implement, and retain participants for the full cine-VR training. Furthermore, participants reported no adverse effects or issues with the cine-VR technology. In total, 3-quarters of the participants completed the pre- and postmeasures for the pilot study. Overall, we observed significant improvements in participants’ awareness of discrimination toward people with disability, self-efficacy to identify and manage elder abuse and neglect, and empathy. These improvements suggest that the cine-VR training program may be an effective teaching modality to educate health professional students about the complexity of care in older adults. More research is needed with a larger, more diverse sample and a proper attention control condition to confirm its effectiveness.

An objective of the cine-VR training program was to raise awareness about discrimination towards aging and disabilities. The cine-VR training portrayed multiple interactions among Mr Chen, his family, and health care providers to raise awareness about discrimination. In one simulation, for instance, Mr Chen’s grandson is depicted yelling “Good morning!” and laughing, a reaction to his grandfather’s hearing loss. Later in the same simulation, his daughter-in-law appears visibly frustrated following an incident of urinary incontinence. These interactions were included to show that sometimes people, including family members, make fun of disabilities or become impatient with people who have a disability. Similarly, the cine-VR training included episodes involving the primary care physician and urgent care physician, directing their questions about Mr Chen’s medical history to his son and daughter-in-law, rather than speaking to Mr Chen directly. This was done intentionally to suggest that the providers did not think Mr Chen was capable of discussing his own health. In addition, we included an insensitive comment from the urgent care physician directed at Mr Chen poking fun of his situation. These scenarios were designed to illustrate various types of discrimination and elicit emotional reactions from the participants. Learning that involves emotions increases memory retention through emotional engagement, enhanced cognitive processing, and memory consolidation [19,20]. The brain tends to prioritize emotionally significant information for long-term memory over neutral information, which may explain the increased awareness of discrimination observed in the findings [21].

The cine-VR training program also emphasized recognizing the signs of elder abuse and neglect, how to report abuse, and the subsequent actions taken after a report is made. Each episode uncovered new signs of elder abuse and neglect involving Mr Chen (eg, withholding fluids throughout the day to prevent urinary incontinence, not refilling Mr Chen’s medications, and making Mr Chen live in the garage while the house is being renovated), alongside behaviors commonly exhibited by abusers. The follow-up debriefs and reflections concentrated on the responsibilities of mandated reporters, the reporting process, and the steps that follow a report. This content likely contributed to the participants’ increased self-efficacy in asking older adults about abuse, ensuring questions asked about abuse are asked in private, providing emotional support to an older adult who tells you about abuse, helping an older adult subjected to abuse to the right authorities, helping an older adult subjected to abuse who does not want to change circumstances, and handling an older adult who denies abuse but you have strong suspicions that they are subjected to abuse. Additional research is needed to confirm if these increases in self-efficacy are sustained over time.

### Comparison With Previous Work

Medical training uses a variety of simulation types to prepare health professional students for clinical practice. The most common types of simulation include manikins and advanced patient simulators, standardized patients, and task trainers [22]. The first manikin-based simulation can be traced back to the 1960s with the development of Resusci Anne for CPR training [23]. Since then, health professional students have used manikins and advanced patient simulators to replicate complex medical scenarios, including cardiac arrest, childbirth delivery, and trauma resuscitation [23]. The advantages of using manikins and advanced patient simulators include a risk-free setting for repeated practice of clinical skills and standardized training experiences [24-26]. Drawbacks of using manikins and advanced patient simulators include the high purchase price, ongoing maintenance costs, and the technological limitations that prevent them from fully replicating the complexity and unpredictability of human behavior [27,28]. Conversely, standardized patients provide a more realistic interaction for students. Standardized patients have been shown to improve communication skills, critical thinking, self-efficacy, and clinical examination skills [29]. Despite training, standardized patients may show individual differences that can lead to variations in role portrayal, impacting the consistency of the simulated learning experience [30]. Unlike manikins, advanced patient simulators, and standardized patients, a task trainer is a specific device designed to help train health professional students on how to perform specific procedures, such as inserting an intravenous line or performing a lumbar puncture. Task trainers allow for focused learning, skill mastery, and standardization of training in a safe learning environment equipped to provide feedback and assessment; however, they are limited in scope, lack human interaction, and require regular maintenance and upkeep to remain functional [22].

Cine-VR builds on these traditional simulation methods by integrating narrative storytelling and interactive visual experiences [13,14,31,32]. Unlike manikins, advanced patient simulators, and task trainers, cine-VR offers an immersive environment that shows real-life scenarios, situational complexity, and human emotions [33]. Furthermore, cine-VR can provide both physical and decision-based feedback through interactive episodes, whereas task trainers only provide physical feedback for procedural skills. Furthermore, standardized patients offer realistic encounters with patients; however, actors can vary in their portrayal of patients, which affects the consistency of the training. Cine-VR can standardize patient interactions while also allowing for variations in patient interactions to balance consistency with the flexibility to adapt to human unpredictability. Altogether, cine-VR incorporates elements from manikins, advanced patient simulators, standardized patients, and task trainers into a single comprehensive training tool [33].

Our cine-VR training program has several advantages compared with other simulation approaches. First, our cine-VR was standardized, safe, and reusable. If needed, participants can repeat the training as often as desired to learn or reinforce knowledge and clinical skills. In addition, participants could practice difficult, high-stakes conversations through our “guided simulations,” without any risk or harm to the patient, Mr Chen. Next, the multisensory experience of cine-VR engages emotional learning. Research on emotional learning shows that it increases long-term memory as well as increased acquisition of skills [34-36]. Finally, cine-VR training offers participants a glimpse into the lives of their patients, thereby increasing their awareness of another person’s feelings and experiences. This form of perspective-taking increases empathy similar to the findings in our pilot study [32,37,38]. A 2017 study by Schutte and Silinovic [39] compared the impact of VR and traditional didactic learning on empathy among undergraduate students. They found participants in the VR group reported greater increases in empathy compared to the didactic presentation group. Thus, cine-VR has the potential to increase empathy in health professional students; more research is needed to assess empathy and other prosocial behaviors (ie, helping, sharing, and comforting) in future research. Considering patients value empathy from their health care professionals, often as much or even more than clinical expertise, simulation training that enhances empathy is crucial [40,41].

### Limitations

Limitations of this study included the small sample, data collected from one site, heterogeneity of health care specialties, selection bias, subject bias, and lack of a control group. We recruited a sample of 65 participants for the training, and of those, 48 completed the pre- and postassessment. While the sample of 48 participants was small, it was sufficient to pilot the measures for our pilot study. Next, we collected data from one university in the Midwest, which limits the generalizability of our findings to all health professional students. Furthermore, participants represented 6 different health professional fields. Geriatric care is inherently interprofessional and typically requires collaboration from multiple health care professionals. However, the small sample size across 6 health professional programs limits the ability to draw conclusions about the cine-VR training program in these fields. In addition, students who volunteered to participate may have been more willing or motivated to participate in this cine-VR training on care for older adults. Thus, the findings may be susceptible to subject bias and social desirability bias. Finally, we did not include a control condition as a comparison group. Future research with a large sample size must include a control condition to determine the effectiveness of the cine-VR training. In future work, we plan to conduct a randomized controlled trial comparing the effectiveness of the cine-VR training to simulated patients. Simulated patients are used extensively in medical training to enhance the educational experience of health professional students; however, simulated patients can be costly and time-consuming. If we demonstrate that the cine-VR training is more effective or equally effective as simulated patients, cine-VR training may be a more sustainable, cost-effective approach to refine health professional students’ clinical and communication skills without the risk of harming real patients.

### Conclusions

We demonstrated that our cine-VR training program was feasible and acceptable to health professional students at our university in the Midwestern United States. Our findings suggest that the training increased awareness of discrimination towards people with disability, improved self-efficacy to identify and manage elder abuse and neglect, and increased empathy. More research is needed to confirm the effectiveness of this cine-VR training program on the complexity of care for older adults. Future research should use a randomized controlled trial to compare the cine-VR to an attention control condition, such as simulated patients. If confirmed, the cine-VR training program may be a new, effective approach to learning about multimorbidity, geriatric syndromes, and biases related to aging.

## Abbreviations

cine-VR: cinematic–virtual reality
VR: virtual reality
